# Ultraviolet Vision and Avoidance of Power Lines in Birds and Mammals

**DOI:** 10.1111/cobi.12262

**Published:** 2014-03-12

**Authors:** Nicholas Tyler, Karl-Arne Stokkan, Chris Hogg, Christian Nellemann, Arnt-Inge Vistnes, Glen Jeffery

**Affiliations:** 1 Centre for Saami Studies, University of TromsøN-9037 Tromsø, Norway; 2 Department of Arctic and Marine Biology, University of TromsøN-9037 Tromsø, Norway; 3 Moorfields Eye HospitalLondon EC1V 2PD, United Kingdom; 4 UNEP GRID ArendalN-2615 Lillehammer, Norway; 5 Department of Physics, University of OsloN-0316 Oslo, Norway; 6 Institute of Ophthalmology, University College LondonLondon EC1V 9EL, United Kingdom

The avoidance by mammals and ground-nesting birds of habitat up to several kilometers from high-voltage power lines is a major consequence of infrastructure development in remote areas, but the behavior is perplexing because suspended cables are neither an impenetrable physical barrier nor associated with human traffic (e.g., Vistnes & Nellemann 2008; Pruett et al. 2009; Degteva & Nellemann 2013). Moreover, avoidance may persist >3 decades after construction (Nellemann et al. 2003; Vistnes et al. 2004), suggesting behavioral reinforcement. Integration of new information on visual function with the characteristics of power line function provides compelling evidence that avoidance may be linked with the ability of animals to detect ultraviolet light (UV).

Ultraviolet discharges on power lines occur both as standing corona along cables and irregular flashes on insulators. The discharge spectrum (200–400 nm; Maruvada 2000) is below the normal lower limit of human vision, UV being attenuated by the human cornea and lens, but in birds, rodents, and reindeer/caribou (*Rangifer tarandus*) (hereafter reindeer) the cornea and lens are UV permissive. The former have specific UV sensitive opsins (Bowmaker 2008) and, hence, power line corona may be assumed visually salient in these. Reindeer have no specific UV opsin, but we obtained robust retinal responses to 330 nm mediated by other opsins (Hogg et al. 2011 and unpublished) and propose that corona flashes are both visually salient and a cause of this species avoiding power lines.

Recent demonstration of UV responses in reindeer retinae was based on electrophysiological corneal recordings (Hogg et al. 2011). These, however, are approximately 3 log units less sensitive than psychophysical measurements of visual perception (Ruseckaite et al. 2011). They demonstrate an ability to see UV discharge but are poor indicators of visual threshold and underestimate visual sensitivity. Furthermore, reindeer and some birds have a reflective surface directly behind the retinal photoreceptors (the tapetum lucidum) which ensures that light not captured as it passes through them is reflected back for a second pass, consequently, increasing retinal sensitivity in dark (i.e., very low light) environments (Johnson 1968). In reindeer, the winter adapted tapetum scatters light among photoreceptors rather than reflecting it which enhances photon capture and increases retinal sensitivity by approximately 3 log units at winter threshold (Stokkan et al. 2013).

Other factors increase the likelihood that reindeer see coronal discharges in the dark. First, retinal sensitivity is maximized in reindeer because their retinae are almost permanently dark adapted during the extended dusk of Arctic winters, and, given that the mammalian visual range is approximately 9 log units, fully dark adapted eyes are capable of responding to the stimulus of a single photon. Second, the reindeer eye is larger than the human eye and thus provides greater image magnification, and the pupil, which dilates to 21 mm compared with approximately 10 mm in humans, is likely to be permanently dilated in winter consequently increasing retinal sensitivity approximately 4-fold. Third, dilation exposes more of the peripheral retina that is sensitive to sudden changes in the visual environment.

The stimulus is also important. Ultraviolet discharge is both strongly (approximately 90%) reflected and scattered by snow. Hence, in a snowy landscape the corona is likely to appear brighter to animals responsive to UV than in conventional imaging which focuses on source discharge. Second, and crucially, the pattern of occurrence of corona flashes is temporally random, which is likely to impede habituation.

These observations constitute a strong argument that reindeer, like birds and rodents, may see corona UV. By extension, we suggest that in darkness these animals see power lines not as dim, passive structures but, rather, as lines of flickering light stretching across the terrain. This does not explain avoidance by daylight or when lines are not transmitting electricity—although, interestingly, electrically earthed cables are more hazardous to galliformes (which detect UV to 355 nm; Lind et al. 2014), perhaps precisely because without corona definition is lost (Bevanger & Brøseth 2001)—but it may be an example of classical conditioning in which the configuration of power lines is associated with events regarded as threatening.
